# Plasma lipoprotein (a) and long-term all-cause mortality in myocardial infarction survivors: a cohort study

**DOI:** 10.1186/s12944-026-03111-0

**Published:** 2026-11-11

**Authors:** Timo Schmitz, Jakob Linseisen, Christa Meisinger

**Affiliations:** Epidemiology, Medical Faculty, University of Augsburg, University Hospital Augsburg, Stenglinstraße 2, 86156 Augsburg, Germany

**Keywords:** Lp(a), Long-term mortality, Acute myocardial infarction, Survival analysis

## Abstract

**Background:**

Elevated concentrations of lipoprotein (a) [Lp(a)] are a well-established risk factor for the development of coronary artery diseases and acute myocardial infarction (AMI). The objective of the present study was to analyze the association between plasma concentrations of Lp(a) and long-term all-cause mortality among individuals with a history of hospitalized AMI.

**Methods:**

The analysis was based on data from 617 patients with a history of AMI recruited by the Augsburg Myocardial Infarction Registry and examined in a follow-up visit within the scope of the KORA Family Heart Study in 1996 and 1997 (median time between AMI and follow-up examination: 6.2 years). In addition, 545 siblings of the patients were included in the study and underwent examination. The examination included venous blood sampling and a patient interview. Plasma concentrations of Lp(a) were measured using a latex-enhanced immunoassay. Information on vital status was obtained in November 2025 in a comprehensive mortality follow-up [median follow-up time: 18.2 years (IQR: 10.8–27.7)]. Follow-up time was calculated from examination date to date of death or censoring. Kaplan-Meier curves were used to display estimated survival probability of Lp(a) high (≥ 50 mg/dL) and low (< 50 mg/dL) group. Multivariable adjusted Cox regression models with restricted cubic splines were used to analyze the association between Lp(a) concentrations and long-term all-cause mortality after AMI.

**Results:**

Kaplan-Meier curves and log rank test (p value: 0.273) demonstrated no differences in long-term all-cause mortality between the high and low Lp(a) group. The multivariable adjusted Cox regression confirmed the absence of a significant association between Lp(a) concentrations and total mortality (HR for the high Lp(a) group: 1.04, 95%CI: 0.84–1.28, p value: 0.734). Several sensitivity analyses, including a mixed model on a sample including siblings of the AMI patients (HR for the high Lp(a) group: 1.09) and a more thoroughly adjusted model including additional potential confounders confirmed the results of the main model.

**Conclusions:**

In this study, no association between elevated Lp(a) and long-term mortality was detected. Due to the observational character of the study, no conclusions about treatment of patients with elevated Lp(a) can be made and results must be regarded as exploratory and hypothesis generating.

**Supplementary Information:**

The online version contains supplementary material available at https://doi.org/10.1186/s12944-026-03111-0.

## Introduction

Coronary artery disease (CAD) and acute myocardial infarction (AMI) are leading causes of death worldwide and provide a major health burden in many countries [1]. In most cases, an AMI occurs as a result of an underlying CAD [2]. The latter describes arteriosclerotic changes in the coronary arteries, which result from chronic inflammatory processes and lipid deposition in the arterial wall and lead to plaque formation [3]. Rupture of such plaques can result in acute ischemia of myocardial vessels and tissue, leading to the occurrence of AMI [2]. There are many known risk factors for CAD, which can be categorized as modifiable (such as smoking, obesity, and hypertension), and non-modifiable (such as family predisposition, male sex, and advanced age) [4]. One important comorbidity is hyperlipidemia, which refers to abnormal concentrations of blood lipids, including triglycerides and cholesterol. While the role of elevated triglycerides in CAD still remains controversial, elevated cholesterol is well established as a major pathophysiological factor in atherosclerosis [5]. Whereas high-density lipoprotein (HDL) is supposed to be protective, high concentrations of low density lipoprotein (LDL) are thought of as a major risk factor which can be treated well with medication [6]. One special LDL is lipoprotein (a) (Lp[a]) [7] which is bound to apolipoprotein (a) [7] and has proinflammatory and atherogenic properties [8]. Elevated Lp(a) levels are a well-known risk factor for cardiovascular diseases (CVD) including CAD and AMI [8]. On the other hand, there is some evidence that very low levels of Lp(a) are associated with an increased risk of type 2 diabetes mellitus [9, 10], highlighting its complex biological role. Lp(a) concentrations are largely genetically determined, remain quite stable throughout life, and are minimally affected by lifestyle [8], making a single lifetime measurement sufficient for risk assessment in most individuals [8, 11]. About one fifth to one fourth of the global population might be affected by elevated levels of Lp(a) whereby women generally tend to have higher Lp(a) levels compared to men [8, 12, 13]. Even though the potentially causal role of Lp(a) in the development of CVD including CAD [12] is largely accepted and well-established, it remains unclear whether Lp(a) is also a risk factor for adverse outcome in patients with established CAD or after (hospitalized) AMI [14]. While some studies suggested a positive association between Lp(a) and increased long-term mortality in these individuals [15, 16], others do not [14, 17, 18]. So, the aim of this study was to analyze the association between Lp(a) levels and long-term all-cause mortality in individuals with a history of hospitalized AMI.

## Materials and methods

### Study population and data collection

The patients included in the present analysis were AMI survivors who were initially recruited by the Augsburg Myocardial Infarction Registry and took part in a follow-up examination named ‘The KORA Family Heart Study’. The registry was established in 1984 and operates using a population-based approach for data collection. Every case of hospitalized myocardial infarction and prehospital death due to CAD is recorded in case the patient was aged 25 to 74 years (the upper age limit existed until 2009) and had its primary residence within the study area of Augsburg (approximately 700,000 inhabitants in 2024) [19]. Detailed information on the registry and its methodology of data collection can be found elsewhere [20]. In the ‘The KORA Family Heart Study’ [21], 641 individuals surviving hospitalized AMI and younger than 60 years were identified by the Augsburg Myocardial Infarction Registry. The study included a follow-up examination of AMI survivors in the years 1996 and 1997 (median time between examination and AMI: 6.2 years). Additionally, a total of 545 siblings without a history of AMI were examined in the same manner as well. Next to a cardiac echocardiography carried out by a single experienced investigator and a two-channel Holter electrocardiogram ECG, the follow-up examination included venous blood sampling and an extensive patient’s interview including questions on sociodemographic information, existing comorbidities, risk factors, and medication. Blood pressure was measured three times on the right arm using a random-zero sphygmomanometer (Hawksley & Sons) after a standardized resting period of at least 5 min while sitting, with the mean of the second and third measurements used for analysis.

The data collection of the Myocardial Infarction Registry and the KORA Family Heart Study have been approved by the Ethics Committee of the Bavarian Medical Association. The Myocardial Infarction Registry Augsburg was registered at the German Register of Clinical Studies (DRKS, project number DRKS00029042). Both studies were carried out in accordance with the principles of the Declaration of Helsinki. All participants gave written informed consent.

### Mortality follow-up

A recent mortality follow-up of all included individuals (AMI patients and siblings) was conducted in 11/2025. Therefore, the relevant information was obtained from the residents’ registration offices in the study region. All individuals still alive at the time of follow-up were censored as of the end of November 2025. If a study participant’s address was unknown or if the participant was listed as “moved to unknown address,” the censoring date used was the date on which the participant’s status could still be verified as “alive”. Unfortunately, cause-specific mortality data were not available in the present study, because the informed consent form signed by the study participants did not include consent for obtaining death certificates or specific cause-of-death information. Therefore, we were not able to obtain cause-specific mortality data.

### Clinical chemistry measurement

Plasma concentrations of Lp(a) were measured in venous blood samples which were obtained at follow-up, directly processed and analyzed. Immuno latex-enhanced immunoassay (Immuno AG) was used for the measurement of Lp(a) levels. The measurements are given as mg/dL and no conversion to nmol/L is given as the conversion differs between individuals and is therefore discouraged [22]. Detailed technical information on the assay, including apo(a) isoform sensitivity, WHO/IFCC traceability, coefficients of variation, and the lower limit of detection, were not available for the measurements. However, this does not affect the internal consistency of Lp(a) measurements within our study. Total cholesterol and supernatant cholesterol concentrations after a precipitation procedure using dextran sulphate (Immuno AG, Heidelberg, Germany) were measured by the CHOD-PAP method (Boehringer Mannheim, Mannheim, Germany with Hitachi 747 instrument). Serum LDL-C levels were calculated as the difference of total cholesterol and supernatant cholesterol. Serum creatinine was measured by a modified Jaffe method and plasma CRP concentrations were measured using a high-sensitivity assay.

### Statistical analysis

Categorical variables were presented as absolute frequencies (percentages) and compared using Chi-square tests. Continuous variables were presented either as mean (standard deviation [SD]) or median (inter-quartile range [IQR]) and compared using Student’s t tests and Mann–Whitney U tests respectively.

#### Survival analysis

First, Kaplan-Meier curves were used to visualize differences in unadjusted long-term all-cause mortality between AMI patients with Lp(a) concentrations of less than 50 mg/dL (low Lp(a) group) and patients with Lp(a) ≥ 50 mg/dL (high Lp(a) group); a log-rank-test was performed to test for significant survival differences. Even though there is no universally accepted consensus on Lp(a) thresholds, ≥ 50 mg/dL is currently proposed as a cutoff for increased risk of CAD and AMI by the American College of Cardiology [23] and the European Atherosclerosis Society [24].

Next, a multivariable adjusted Cox regression was calculated to analyze the association between Lp(a) plasma concentration (exposure) and long-term all-cause mortality. To capture any non-linearity, restricted cubic splines with 4 knots were used for the exposure variable. The model was further adjusted for the following covariables: sex (male, female), age (in years), diabetes mellitus (yes/no), left-ventricular ejection fraction (LVEF, Teichholz formula), smoking status (current/ex/never smoker), medication with lipid lowering drugs (yes/no), and BMI (kg/m²). BMI was included in the model as both a linear and a squared term, since the association between BMI and long-term mortality after AMI was reported to be nonlinear (obesity paradox) [25]. Because enrolment was conditional on survival to the 1996–97 examination — requiring, for each patient, survival for a variable interval after AMI (median 6.2 years, interquartiles range: 2.3 to 9.2 years) — the effect we estimate is conditioned on surviving to examination. Our estimand is the association of Lp(a) with mortality among those who survived to examination, not among all patients from AMI onset.

#### Additional analysis

Several variables were considered potential confounders, but their role as confounders versus mediators could not be unequivocally established based on current knowledge. These variables included LDL-C, creatinine, CRP and arterial hypertension. Creatinine and arterial hypertension may act as confounders but could also be related to pathways through which Lp(a) is associated with adverse cardiovascular outcomes and mortality. In particular, renal function has been shown to modify the association between Lp(a) and cardiovascular outcomes and has been proposed as a potential mediator of this relationship [26]. Although hypertension may act as a confounder, its relationship with Lp(a) and cardiovascular outcomes appears to be more complex, with evidence suggesting that hypertension may modify the association between Lp(a) and cardiovascular disease. Given the uncertainty regarding its causal position in the Lp(a)–mortality relationship, we did not include hypertension in the main model, as adjustment could potentially attenuate the total association [27]. Furthermore, we could not establish LDL-C as an unequivocal confounder. Adjustment for LDL is potentially problematic because of the intrinsic relationship between LDL-C and Lp(a), including collinearity and possible attenuation of the estimated Lp(a)-associated risk [28]. CRP was considered more plausibly a mediator than a confounder and was therefore not included in the comprehensively adjusted model. We consequently calculated an additional Cox model including LDL-C, creatinine, and arterial hypertension. This model was considered a sensitivity analysis rather than a more appropriate or superior model, given the potential risk of overadjustment for mediators and competing lipid risk factors.

Then we tested the original model for a significant multiplicative interaction between Lp(a) concentrations and diabetes (categorical). Prior studies indicated that LDL and CRP might be effect modifiers of the association between Lp(a) and mortality, therefore we also tested for multiplicative interactions between Lp(a) and LDL [29, 30] and CRP [30] respectively. Moreover, we also tested for additive interactions between Lp(a) [natural cubic splines with 5 degrees of freedom, knots: 2 boundary knots at minimum/maximum values and four interior knots at 20th, 40th, 60th and 80th percentile] and LDL and CRP (continuous variables), respectively, by calculating Aalen additive hazards model (using the *aalen*-function of the R package *timereg*). Additionally, we calculated RERI values for the Cox model using dichotomized Lp(a) [cut-off 50 mg/dL] and dichotomized LDL and CRP [using median as cut-off] respectively. The RERI values were calculated using the R´s *interactionR* package.

Next, we modeled a Cox model just like the main model but excluding patients who died within the first two years after follow-up examination to remove post-examination early deaths, address​ reverse causation and assess early-outcome robustness (early-death exclusion sensitivity analysis). Then we evaluated whether left-truncation of data affected the observed associations. Therefore, we calculated two additional Cox models. One model using age-as-timescale; specifically an attained-age delayed-entry model with entry at examination age (see supplementary results 1). A second model was then calculated as a time-since-AMI delayed-entry model, see also supplementary results 1. We also calculated both models (attained-age delayed-entry model and time-since-AMI delayed-entry model) using the dichotomized Lp(a) variable (cut-off: 50 mg/dL). We calculated each model once including age at AMI as covariable and once without age at AMI.

Moreover, we calculated a Cox model using the dichotomized Lp(a) instead of continuous Lp(a) concentrations. The model was adjusted as described above. Furthermore, the association between Lp(a) [dichotomized] and all-cause mortality was analyzed on the sample including the relatives of the AMI patients. As related individuals form their own cluster (clustered data), survival mixed models were calculated using R´s coxme-package [31]. The mixed model was not calculated to perform within-family comparisons but rather as a mixed-case control analysis. The mixed model was adjusted for sex, age, diabetes mellitus, LVEF, smoking status, medication with lipid lowering drugs, BMI (linear and squared term), and history of AMI (yes/no). We also tested the mixed survival model for a significant interaction with history of AMI. Finally, we calculated a model exactly as the main model to analyze the association between Lp(a) and long-term mortality only in a the sample of siblings without a history of hospitalized AMI.

#### Model assumptions

The proportional hazards assumption was assessed for both categorical and continuous covariables. For categorical variables, the proportional hazard assumption was checked graphically using log(-log(survival)) plots. Furthermore, we plotted the Schoenfeld residuals against time and tested for a significant correlation between residuals and time. Multicollinearity among the independent variables was ruled out via calculation of the Variance inflation factor (VIF).

To evaluate the ability to find significant differences, we performed a post-hoc power analysis using different assumptions for HR and r² (the percent of variation in the predictor of interest explained by other covariates expected to be adjusted for in the Cox regression model).

For all regression analyses, a complete case analysis was performed, and no imputation of data was conducted (e.g. multiple imputation by chained equations). In supplementary results 2 we display the numbers and percentages of missing values for each variable. Given the small number of missing observations across the covariables, a complete-case analysis can be considered as a reasonable approach. P values below 0.05 were considered statistically significant. All analyses were conducted using R version 4.4.3.

## Results

### Baseline characteristics

**Fig. 1:**
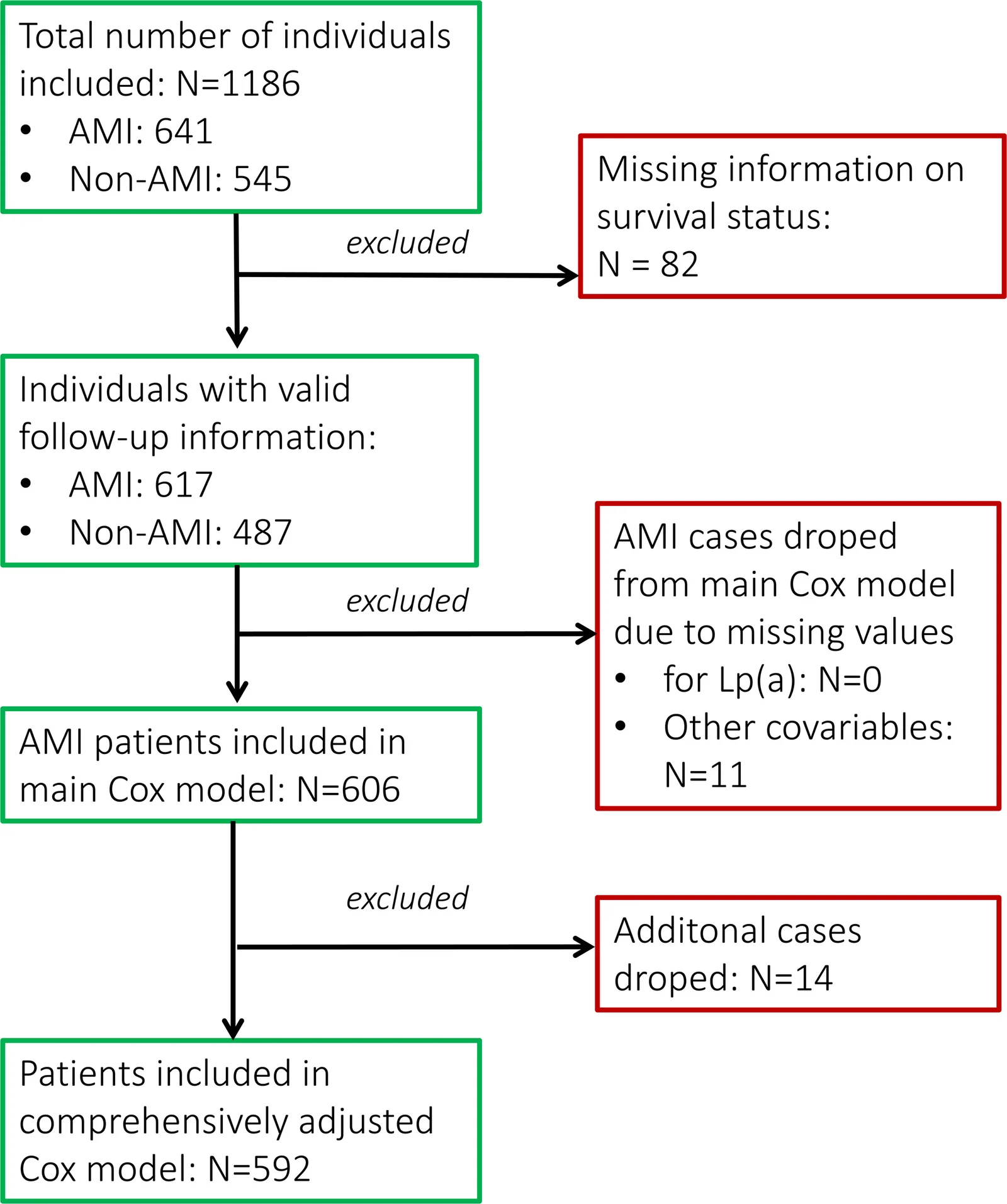
Flow chart of all inclusions and exclusions and final study sample

Excluding 82 patients with missing information on survival status left 617 AMI patients and 487 siblings for the statistical analysis, see Fig. 1. The mean age of the 617 AMI patients included in the present analysis was 56.6 years and thereby slightly older than the included relatives, whose mean age was 55.0 years (Table 1). While the majority of AMI patients were male (87.2%), the group of relatives had an equal representation of both genders. Median Lp(a) concentrations were significantly higher in the AMI group compared to their relatives (17.0 vs. 13.0 mg/dL, p value: 0.035). Among the AMI patients, 167 (27.1%) had Lp(a) concentrations of 50 mg/dL or higher. These patients were younger than those with lower Lp(a) concentrations (mean age in years: 55.5 vs. 57.0, p value: 0.020) and were significantly less likely to have a diagnosis of diabetes mellitus (7.8% vs. 17.6%, p value: 0.004). The two Lp(a) groups did not differ significantly regarding sex, BMI, smoking status, blood pressure, LDL cholesterol, creatinine, CRP or the use of lipid lowering drugs, statins, beta blockers, platelet aggregation inhibitors, and ACE inhibitors.

**Table 1:** Baseline characteristics of the total sample and stratified for myocardial infarction (no/yes). The group of individuals with prior myocardial infarction are further displayed stratified for Lp(a) high (≥ 50 mg/dL) and low (< 50 mg/dL)

	Total sample					AMI sample		
	Total sample (*N* = 1104)	N*	No AMI (*N* = 487)	AMI (*N* = 617)	*P*-Value	Lp(a) ≥ 50 mg/dL (*N* = 167)	Lp(a) < 50 mg/dL (*N* = 450)	*P*-Value
Died during follow-up	697 (63.1)	1104	219 (45.0)	478 (77.5)	< 0.001	125 (74.9)	353 (78.4)	0.400
Incidence rate (deaths per 1000 person years)	30.7	-	18.8	43.4	-	40.5	44.4	-
Male	772 (69.9)	1104	234 (48.0)	538 (87.2)	< 0.001	141 (84.4)	397 (88.2)	0.264
Age (mean, SD)	55.9 (8.0)	1104	55.0 (8.8)	56.6 (7.2)	0.001	55.5 (7.5)	57.0 (7.1)	0.020
Lp(a) (mg/dL)	15.0 (6.0–48.2)	1104	13.0 (6.0–39.5)	17.0 (6.0–56.0)	0.035	91.0 (70.0–114.0)	10.0 (4.0–20.0)	< 0.001
Lp(a) ≥ 50 mg/dL	273 (24.7)	1104	106 (21.8)	167 (27.1)	0.050	-	-	-
BMI (kg/m²)	27.7 (25.4–30.3)	1101	27.1 (24.9–29.8)	28.1 (25.8–30.6)	< 0.001	27.6 (25.2–30.6)	28.3 (25.9–30.6)	0.115
Diabetes mellitus	122 (11.1)	1101	30 (6.2)	92 (15.0)	< 0.001	13 (7.8)	79 (17.6)	0.004
Smoking status		1102			< 0.001			0.142
Never smoker	361 (32.8)		226 (46.4)	135 (22.0)		39 (23.5)	96 (21.4)	
Ex smoker	531 (48.2)		141 (29.0)	390 (63.4)		96 (57.8)	294 (65.5)	
Current smoker	210 (19.1)		120 (24.6)	90 (14.6)		31 (18.7)	59 (13.1)	
LVEF**	54.0 (46.0–61.0)	1092	58.0 (50.0–64.0)	50.0 (44.0–58.0)	< 0.001	51.0 (44.0–59.0)	50.0 (44.0–58.0)	0.571
Low density lipoprotein (LDL) cholesterol (mg/dL)***	135.0 (110.0–162.0)	1089	139.5 (113.0–167.2)	131.0 (106.0–158.0)	0.001	136.5 (112.8–161.2)	128.0 (104.0–157.0)	0.08
C-reactive Protein (CRP, mg/L)***	1.8 (1.0–3.7)	1090	1.6 (0.8–3.3)	2.0 (1.1–3.9)	0.003	1.8 (1.0–3.9)	2.0 (1.1–3.9)	0.731
Creatinine (mg/dL)***	0.9 (0.8–1.0)	1100	0.8 (0.7–0.9)	0.9 (0.8–1.0)	< 0.001	0.9 (0.8–1.1)	0.9 (0.8-1.0)	0.319
Systolic blood pressure***	133.0 (123.0–146.0)	1104	136.0 (126.0–149.5)	130.0 (120.0–143.0)	< 0.001	129.0 (120.0–141.0)	131.0 (121.0–143.8)	0.376
Diastolic blood pressure***	85.0 (79.0–91.0)	1104	86.0 (80.0–93.0)	84.0 (78.0–90.0)	0.001	85.0 (78.0–90.0)	83.0 (78.0–90.0)	0.387
Medication at baseline								
Lipid lowering drugs	292 (26.5)	1103	35 (7.2)	257 (41.7)	< 0.001	78 (46.7)	179 (39.8)	0.145
Statins	228 (20.7)	1103	23 (4.7)	205 (33.2)	< 0.001	65 (38.9)	140 (31.1)	0.083
Beta blockers	453 (41.1)	1103	59 (12.1)	394 (63.9)	< 0.001	107 (64.1)	287 (63.8)	1
Platelet aggregation inhibitors	550 (49.9)	1103	44 (9.1)	506 (82.0)	< 0.001	135 (80.8)	371 (82.4)	0.731
ACE inhibitors	219 (19.9)	1103	48 (9.9)	171 (27.7)	< 0.001	40 (24.0)	131 (29.1)	0.242

* Number of cases with valid information for each variable

** left-ventricular ejection fraction (Teichholz formula)

*** measured at follow-up examination

Supplementary table S1 displays characteristics of the individuals included and excluded due to unknown survival status. For AMI patients, excluded persons (*N* = 24) were significantly younger at follow-up examination and had a significantly higher BMI. There were no significant differences regarding sex, Lp(a) concentrations, other comorbidities or medication.

### Survival analyses

The median follow up time (beginning at follow-up examination) was 18.2 years (IQR: 10.8–27.7) for individuals with a history of hospitalized AMI and 23.1 years (IQR: 13.7–28.7) for the total sample. For AMI patients, the incidence rate (deaths per 1000 person years) was 43.4 and for the siblings without history of AMI 18.8 (incidence rate ratio: 2.31). In the group of AMI patients with Lp(a) concentrations ≥ 50 mg/dl, the incidence rate was 40.5 compared to 44.4 in the group with low Lp(a) concentrations, which gives a rate ratio of 0.91.

**Fig. 2:**
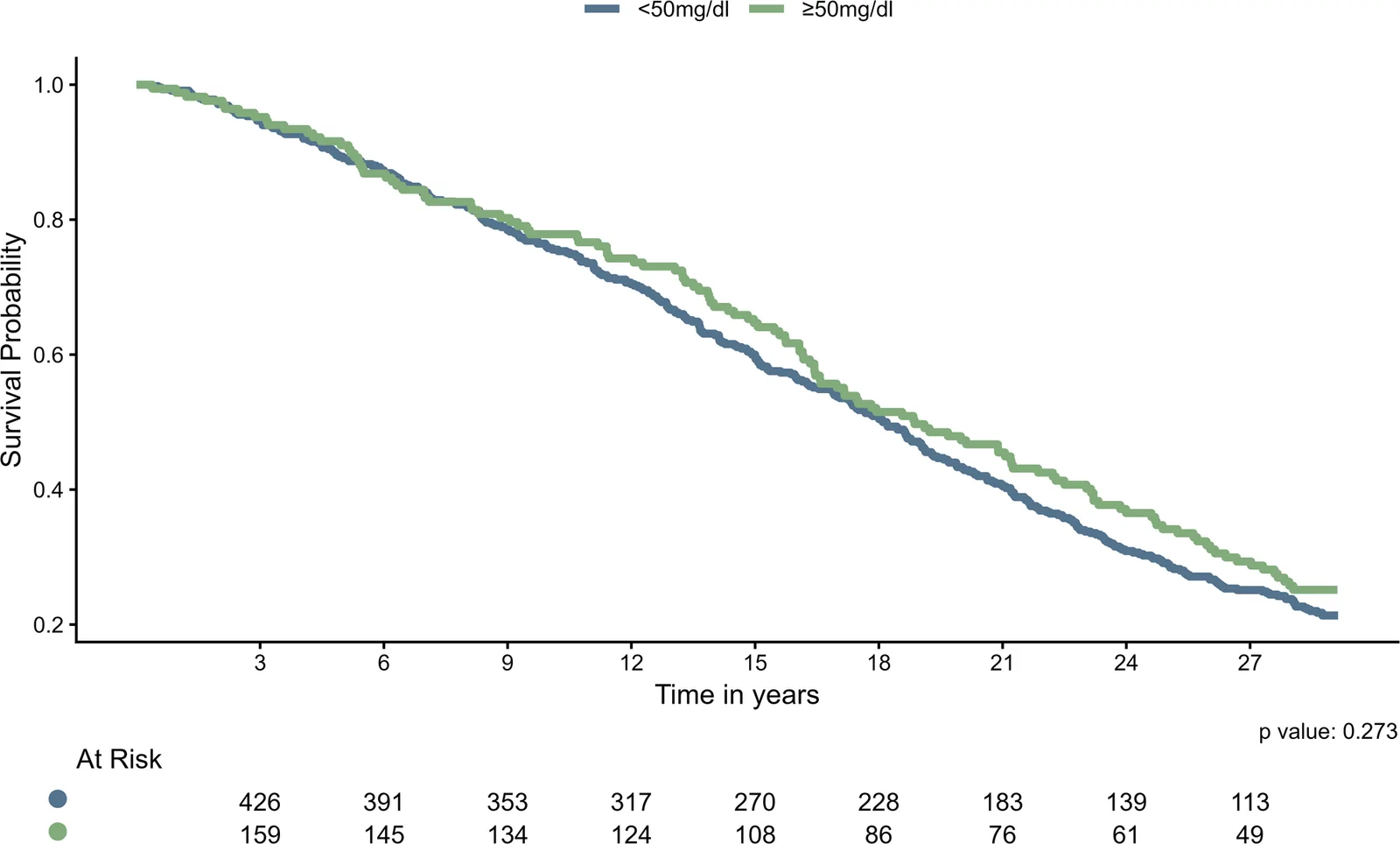
Survival curves of acute myocardial infarction patients stratified for Lp(a) concentration (< and ≥ 50 mg/dL) and compared using log-rank test

**Fig. 3:**
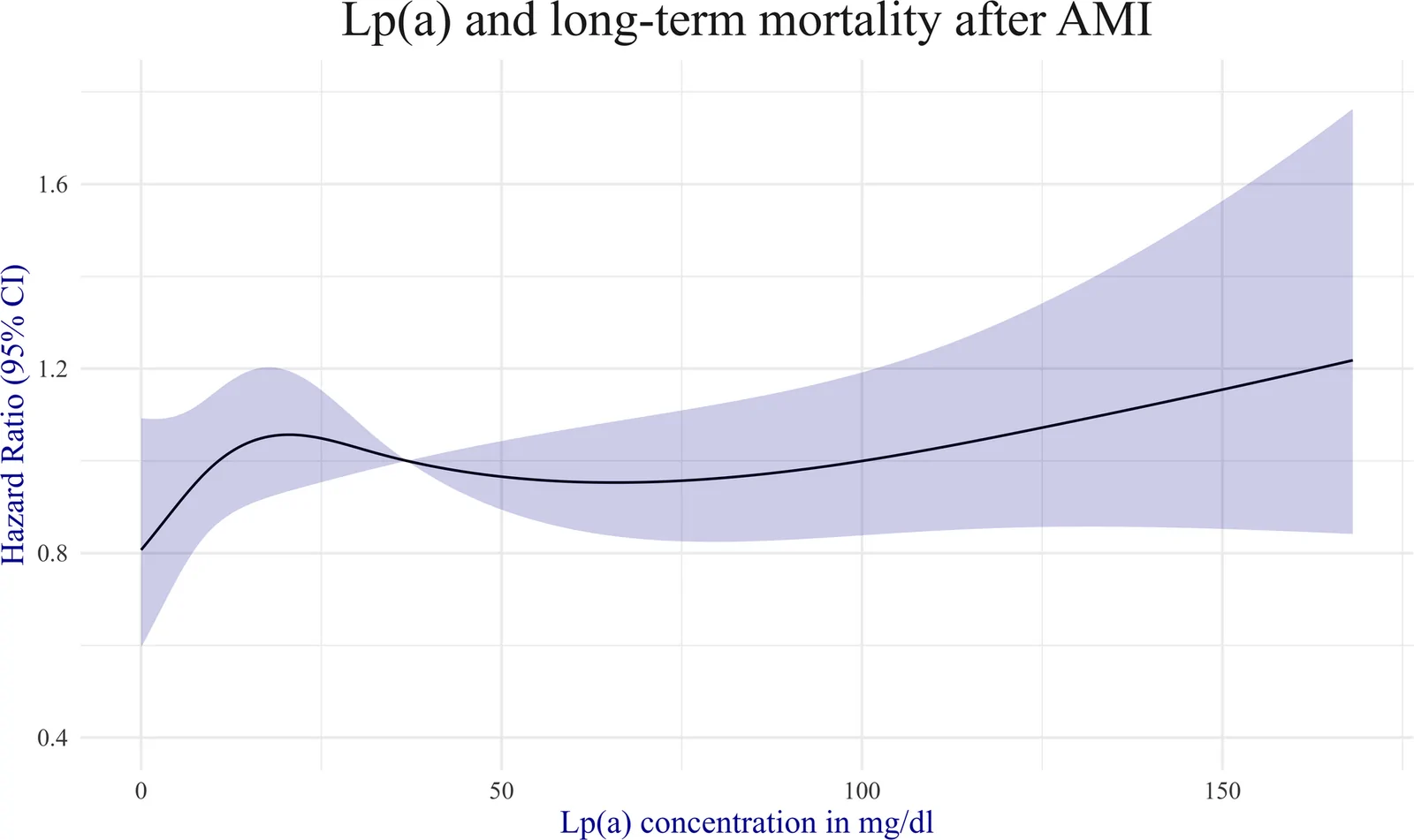
Association between Lp(a) and long-term mortality after AMI. For the Cox regression model, restricted cubic splines with 4 knots (knot placement: 1, 9, 32.4, 124.6 mg/dL) were used and the model was adjusted for sex, age, BMI (linear and squared term), smoking, diabetes, left-ventricular ejection fraction, and intake of lipid lowering drugs. The reference concentration was 36.9 mg/dL (mean value). The numer of cases was 606 with 467 events

Figure 2 shows the Kaplan-Meier curves for AMI patients stratified by Lp(a) ≥ 50 mg/dL and Lp(a) < 50 mg/dL. The survival curves remain closely aligned throughout the entire follow-up period of about 27 years, and the log-rank test reveals no significant difference (p value: 0.273). Figure 3 displays the association between continuous Lp(a) concentrations and long-term all-cause mortality, modeled by Cox regression using restricted cubic splines (total cases included: *N* = 606, number of deaths: *N* = 467); no significant association was found. Results of the checks for model assumptions are presented in supplementary results 2. The more comprehensively adjusted model yielded very similar results, see supplementary figure S1. Excluding patients who died within the first two years after the follow-up examniation (supplementary figure S2) did not change the results substantially indicating that reverse causality did not majorly affect results. Likewise, the Cox model using age-as-timescale (attained-age delayed-entry model, see supplementary figure S3) and the time-since-AMI delayed-entry model (supplementary figure S4) did not materially affect the results suggesting that left truncation of data did not affect results in a relevant manner. In supplementary results 1 the results of the 5 different models are presented each using dichotomized Lp(a) as exposure: the attained-age delayed-entry models (adjusted and not adjusted for age at AMI) and the time-since-AMI delayed-entry model adjusted for age at AMI revealed comparable results to those of the main model (time since examination). The time-since-AMI delayed-entry model not adjusted for age at AMI showed a non-significant HR of 0.93 compared to the non-significant HR of 1.04 of the main model. Moreover, there was no significant multiplicative interaction between Lp(a) concentrations and diabetes, see supplementary text 1. Likewise, no significant multiplicative interactions were found for LDL (continuous), see also supplementary text 1. There was a significant multiplicative interaction between Lp(a) and continuous CRP. However, only the first of three interaction coefficients was significant and the others alternating in sign, so the interaction lacks a clear and explicit direction. Supplementary figure S5 and S6 display the results for Cox models including a multiplicative interaction between Lp(a) and quartiles of LDL and CRP respectively. Moreover, the Aalen additive hazards models revealed non-significant results with ß-coefficients close to 0 for the interaction terms between Lp(a) [natural cubic splines with 5 degrees of freedom] and continuous LDL and CRP respectively, see supplementary text 1. The absence of additive interactions was also confirmed by RERI values close to 0 for both LDL and CRP, supplementary text 1.

Using 50 mg/dL as a cut-off value revealed no significant differences in long-term mortality between individuals below (reference group) and above the threshold of 50 mg/dL (HR: 1.04, 95%CI: 0.84–1.28, p value: 0.734). Consequently, the point-estimate E-value on the relative risk scale would be 1.20 and 95% CI-E value is 1 (non-rare outcome). Assuming a potential true estimate of HR = 1.2, the corresponding point-estimate E-Value would be 1.44.

The mixed model analyzing the association in the total sample (mixed case-control approach including AMI cases and their siblings) confirmed the main results, showing no significant increase in long-term mortality for individuals with Lp(a) values ≥ 50 mg/dL (HR: 1.09, 95%CI: 0.91–1.31, p value: 0.356). The model revealed no significant interaction between Lp(a) and history of AMI. Finally, supplementary figure S7 reveals no significant association between Lp(a) concentrations and long-term mortality in the group of non-AMI individuals.

The post-hoc power calculation, calculated during peer review, revealed a high power of 77.9% and more if a HR of at least 1.4 was assumed, see supplementary table S2. In case of a true HR of 1.2, the study would lack the power to find significant differences (less than 50% regardless of r²). The 95%CI of the dichotomized Cox models indicates that such an effect size (HR = 1.2) is statistically not implausible, so our data does not exclude a range of possible associations.

## Discussion

Despite strong evidence that Lp(a) predicts incident atherosclerotic cardiovascular disease, this cohort with exceptionally long follow-up provides little evidence that Lp(a) predicts long-term mortality among AMI survivors. Inclusion of siblings, who share part of their genetic background and environmental exposures, yielded similar results and therefore supports the robustness of the findings.

Although substantial evidence from multicenter studies, systematic reviews and meta-analyses supports an association between elevated Lp(a) levels and an increased risk of cardiovascular diseases [32, 33], its prognostic value after established cardiovascular diseases (CVD) events remain less clear. A review from 2023 stated that Lp(a) is associated with increased all-cause and CVD mortality in both the general population and patients with pre-existing CVD [16]. Consistent with this, Farnier et al. demonstrated that elevated Lp(a) levels were associated with both increased in-hospital mortality in AMI patients and greater CAD severity in patients presenting with AMI [15]. However, the association between Lp(a) and long-term mortality appears to be complex. For example, a very recent study based on data from the Third National Health and Nutrition Examination Survey (NHANES III) found a significant association between elevated Lp(a) and long-term mortality in individuals with a particularly high baseline risk for cardiovascular diseases, however no significant association was found for persons with low cardiovascular risk [34]. Likewise, the prognostic significance of Lp(a) in patients with established CVD remains inconsistent across studies. Several investigations failed to demonstrate an association between elevated Lp(a) concentrations and mortality in either population-based cohorts [32] or patients with CAD [14] or previous AMI [18]. For example, Zhang et al. identified a significant association between Lp(a) levels and left ventricular ejection fraction (LVEF) in AMI patients. However, while LVEF also predicted all-cause mortality after AMI, Lp(a)​ did not independently predict​ all-cause mortality [18]. Similarly, a Chinese study of patients who underwent PCI confirmed an association between Lp(a) and severity of CAD [17], which is in accordance with the finding of Farnier et al. [15]. Yet elevated Lp(a) was not associated with adverse long-term cardiovascular outcomes after PCI [17].

We observed a nominally significant multiplicative interaction between Lp(a) and CRP, which should be interpreted cautiously. Of the three CRP spline coefficients, only the first had a p-value clearly below 0.05 (*p* = 0.012), while the others were borderline (*p* = 0.050 and *p* = 0.057) and alternated in sign. No evidence of additive interaction was found. Given the number of interaction analyses performed, this isolated finding may be due to chance. Our results also do not clearly reproduce those of Li et al. [30], although direct comparison is limited because we examined separate two-way interactions rather than a three-way interaction of Lp(a), LDL cholesterol, and CRP. Overall, our findings provide no robust evidence of an interaction between Lp(a) and CRP in relation to long-term mortality.

In conclusion, there is strong evidence that Lp(a) plays a major role in the development of CVD and CAD; however, it may not be a useful predictor and risk factor of mortality once these diseases are established. The results of the present study support this perspective, and no significant association was found even decades after AMI. To the best of our knowledge, no previous studies have investigated the association between Lp(a) and mortality after AMI with a follow-up period of up to 27 years.

Several factors may explain the absence of an association between Lp(a) and long-term mortality in AMI survivors. First, Lp(a) might play a more prominent role in the initiation and progression of atherosclerotic disease than in determining prognosis after CAD is established. Additionally, contemporary secondary and tertiary prevention strategies - including intense lipid-lowering therapy, antiplatelet treatment, and lifestyle modification—may attenuate the impact of elevated Lp(a) on long-term outcomes. The study’s inclusion of only patients who survived the acute phase of AMI may have introduced survivorship bias, and competing causes of death over the extended follow-up period could have diluted any association of elevated Lp(a) with increased mortality. Finally, Lp(a) may be more strongly associated with recurrent cardiovascular events or cardiovascular mortality than with all-cause mortality, which was the endpoint assessed in this study.

### Clinical implications

Currently there is no approved and commonly used specific medication to lower circulating Lp(a) concentrations. Treatment mainly focuses on reducing other cardiovascular risk factors, particularly LDL cholesterol [8]. According to the guidelines, statin medication is indicated after AMI [35]. Targeted LDL values vary depending on the patient’s risk profile, but after AMI the aim is usually to get below 55 mg/dL and achieve a reduction of ≥ 50% compared to baseline [35]. Even though reduction of total LDL cholesterol is unquestioned, there is some discussion whether statin medication increases Lp(a) concentrations [36, 37], however cardiovascular benefits of statin treatment are supposed to outweigh the potential risk of increased Lp(a) [8]. Of note, recently, several new treatments using apo(a)-targeting drugs have been developed to reduce Lp(a) levels. Ongoing large-scale phase 3 cardiovascular outcome studies are now investigating whether these marked reductions in Lp(a) translate into lower rates of atherosclerotic cardiovascular events, potentially establishing apo(a)-targeted therapy as a novel strategy for residual cardiovascular risk reduction [38].

Based on observational data, no conclusions on causal relationships can be drawn and therefore no direct clinical implications regarding treatment or prevention measures in the aftercare of patients after hospitalized AMI should be made based on the results of the present study. Further studies, especially clinical trials, are necessary to make reliable statements about clinical implications.

### Strengths and limitations

The present analysis was based on 617 individuals with AMI recruited by the population-based Augsburg Myocardial Infarction Registry and 487 siblings without a history of AMI. All study participants were examined within the scope of the ‘KORA Family Heart Study’. Venous blood samples were taken during follow-up and processed in a standardized manner to guarantee highest quality. Comprehensive information was available for every included individual which allowed a detailed characterization of the study sample and a precise confounder adjustment in the regression analyses. A mortality follow-up of up to 27 years is a rarity and makes this study stand out in particular.

However, there are some important limitations to mention. First, the observational character of the study does not allow for conclusions about causal relationships. Although the regression models were extensively adjusted for potential confounders, the possibility of residual or unmeasured confounding cannot be excluded. For example, no information on acute interventions such as Percutaneous Coronary Intervention or bypass surgery was available. Moreover, in case of small effect sizes (HR of 1.2 for dichotomized Lp(a)) the study sample would be insufficient to reliably detect significant differences. Only AMI patients younger than 60 years were included in the study, so the results may not be applicable to older age groups. Since only individuals who survived until the ‘KORA Family Heart Study’ examination were included (prevalent-cohort selection), survivorship bias cannot be excluded. In contrast to unmeasured confounding bias, which can be quantified by calculating E values, survivorship bias cannot be quantified explicitly. Patients with very high Lp(a) concentrations and poor early prognosis may have died before study enrollment, potentially attenuating any association with long-term mortality. Furthermore, Lp(a) was measured only once at baseline examination. Only data on all-cause rather than cardiovascular mortality were available. Furthermore, LVEF was estimated using the Teichholz formula. This method may be subject to systematic measurement error in patients with regional or segmental wall-motion abnormalities, which are common after AMI. Therefore, the estimated LVEF may not accurately reflect global left ventricular systolic function in all patients. Unfortunately, the individual-level data available for our analysis contains information on the event status and censoring, but not on the specific reasons for censoring, such as moving to an unknown address. We were therefore unable to determine the number or proportion of participants censored for this specific reason. Also, no information on ethnicity was collected. Paré et al. reported major differences in Lp(a) concentrations and isoforms among different ethnicities [39], and therefore the findings may not be generalizable to other ethnic groups and countries.

## Conclusions

The present study found no significant association between elevated Lp(a) concentrations and increased long-term all-cause mortality among AMI survivors. Moreover, we found no indication of elevated risk in a study sample that included relatives of the examined AMI patients. Due to the observational character of the study, no direct clinical implications can be drawn. Further studies are warranted to determine whether Lp(a) predicts other clinically relevant outcomes, such as recurrent cardiovascular events.

## Supplementary Information

Below is the link to the electronic supplementary material.

Supplementary material 1

## Data Availability

The datasets generated during and/or analyzed in the current study are not publicly available due to data protection aspects but are available in an anonymized form from the corresponding author on reasonable request. Requests to access these datasets should be directed to TS, timo.schmitz@med.uni-augsburg.de.
