# Supplementary material for: Plasma lipoprotein (a) and long-term all-cause mortality in myocardial infarction survivors: a cohort study
Source: Lipids Health Dis. 2026 Nov 11;25:227. doi: 10.1186/s12944-026-03111-0 (PMC13769197; doi:10.1186/s12944-026-03111-0)
Supplement: Supplementary file 1 [file 12944_2026_3111_MOESM1_ESM.pdf]

Supplements

Title: Plasma lipoprotein (a) and long-term all-cause mortality in myocardial infarction survivors: a cohort study

Supplementary results 1:

To address a potential effect of left-truncation, additional COX models with alternative time-scales were calculated. The first model was an **attained-age delayed-entry model**. The following calculations were performed using R statistic software:

```
cph(Surv(tstart, tstop, status) ~ rcs(lpa,4) + age_at_AMI + sex + diabetes +
        smoking + bmi + I(bmi^2)+ lipid_lowering_drugs + LVEF, data = data)
# tstart: age at follow-up
# tstop: age at censoring or death
# status: censoring (=0) or death (=1)
```

The second model was a **time-since-AMI delayed-entry model** using the following syntax:

```
cph(Surv(tstart, tstop, status) ~ rcs(lpa,4) + age_at_AMI + sex + diabetes +
        smoking + bmi + I(bmi^2)+ lipid_lowering_drugs + LVEF, data = data)
# tstart: time between AMI and follow-up
# tstop: time between AMI and censoring/death
# status: censoring (=0) or death (=1)
```

In addition, we calculated the alternative models (time-since-AMI delayed-entry model and attained-age delayed-entry model) using dichotomized Lp(a) (cut-off 50 mg/dL) and once including age at AMI as a confounder and once without. All model results are displayed in the subsequent table:

Model	HR (95%CI)	P value	N/events	Schoenfeld test p value*
Main model (time since examination)	1.04 (0.84-1.28)	0.734	606/467	0.036
Attained-age delayed-entry model **not** adjusted for age at AMI	1.04 (0.84-1.28)	0.736	606/467	0.200
Attained-age delayed-entry model adjusted for age at AMI	1.02 (0.82-1.26)	0.887	589/450	0.034
Time-since-AMI delayed-entry model **not** adjusted for age at AMI	0.93 (0.75-1.16)	0.535	589/450	0.087
Time-since-AMI delayed-entry model adjusted for age at AMI	1.01 (0.82-1.26)	0.906	589/450	0.050

* global p value derived from the *cox.zph* function of the R *survival* package.

Schoenfeld residual p values for all variables of the models presented in the table above:

	Main model (time since examination)	Attained-age delayed-entry model **not** adjusted for age at AMI	Attained-age delayed-entry model adjusted for age at AMI	Time-since-AMI delayed-entry model **not** adjusted for age at AMI	Time-since-AMI delayed-entry model adjusted for age at AMI
Lp(a) [dichotomized]	0.829	0.325	0.341	0.819	0.806
Age at follow-up	0.520	-	-	-	-
Age at AMI	-	-	0.021	-	0.032
Sex	0.949	0.521	0.684	0.970	0.878
Diabetes	0.105	0.770	0.613	0.139	0.212
Smoking	0.874	0.891	0.943	0.900	0.870
BMI	0.031	0.389	0.554	0.186	0.221
BMI^2	0.035	0.494	0.672	0.212	0.258
LVEF	0.014	0.126	0.142	0.089	0.119
Lipid lowering drugs	0.045	0.010	0.004	0.013	0.011

Supplementary results 2: Model assumptions and results of the main Cox regression model

Restricted cubic splines with 4 knots:

	knot 1	knot 2	knot 3	knot 4
quantile	5th	35th	65th	95th
Lp(a) values (mg/dL)	1	9	32.4	124.6

Results of the main Cox model

- Number of observations: 606
- Number of events: 467
- Harrells C-index: 0.6637

	ß coefficient	Hazard Ratio (95% CI)	P value
Lp(a) (1)	0.0233	1.02 (0.99-1.05)	0.114
Lp(a) (2)	-0.5962	0.55 (0.26-1.17)	0.122
Lp(a) (3)	0.8171	2.26 (0.81-6.36)	0.121
age	0.0683	1.07 (1.06-1.09)	<0.001
sex - female	-0.2445	0.78 (0.58-1.05)	0.104
diabetes	0.6284	1.87 (1.45-2.42)	<0.001
smoker	0.8063	2.24 (1.64-3.05)	<0.001
ex-smoker	0.093	1.10 (0.87-1.39)	0.443
bmi	-0.0933	0.91 (0.78-1.06)	0.233
bmi^2	0.002	1.00 (1.00-1.00)	0.109
lipid lowering drugs	-0.2428	0.78 (0.65-0.95)	0.011
LVEF	-0.0145	0.99 (0.98-0.99)	0.001

Hazard ratios for selected Lp(a) values

Lp(a) concentration (mg/dL)	HR
20	1.06 (0.93-1.20)
40	0.99 (0.97-1.01)
60	0.95 (0.85-1.07)
80	0.96 (0.82-1.12)
100	1.00 (0.84-1.19)
120	1.06 (0.85-1.31)
140	1.12 (0.86-1.47)

Variance inflation factor

	VIF	
	**main model**	**reduced model***
lp(a) linear	-	1.1
Lp(a) – rest. cubic splines	1.1	-
age	1.1	1.0
sex	1.1	1.1
diabetes	1.1	1.1
smoking	1.2	1.1
BMI	44.7	1.1
BMI^2	44.5	-
lipid lowering drugs	1.0	1.0
LVEF	1.1	1.0

*model including linear Lp(a) and linear BMI to assess true multicollinearity between the covariables

Number of cases and percentages with missing values in covariables included int the main Cox model and the comprehensively adjusted model:

	Main model	Comprehensive model
**Lp(a)**	0 (0%)	0 (0%)
**Age**	0 (0%)	0 (0%)
**Sex**	0 (0%)	0 (0%)
**Diabetes**	2 (0.3%)	2 (0.3%)
**Smoking status**	2 (0.3%)	2 (0.3%)
**BMI**	1 (0.2%)	1 (0.2%)
**Lipid lowering drugs**	0 (0%)	0 (0%)
**LVEF**	8 (1.3%)	-
**Creatinine**	-	3 (0.5%)
**LDL cholesterol**	-	12 (1.9%)
**Hypertension**	-	2 (0.3%)

Table SR1: Schoenfeld-Residuals (main model).

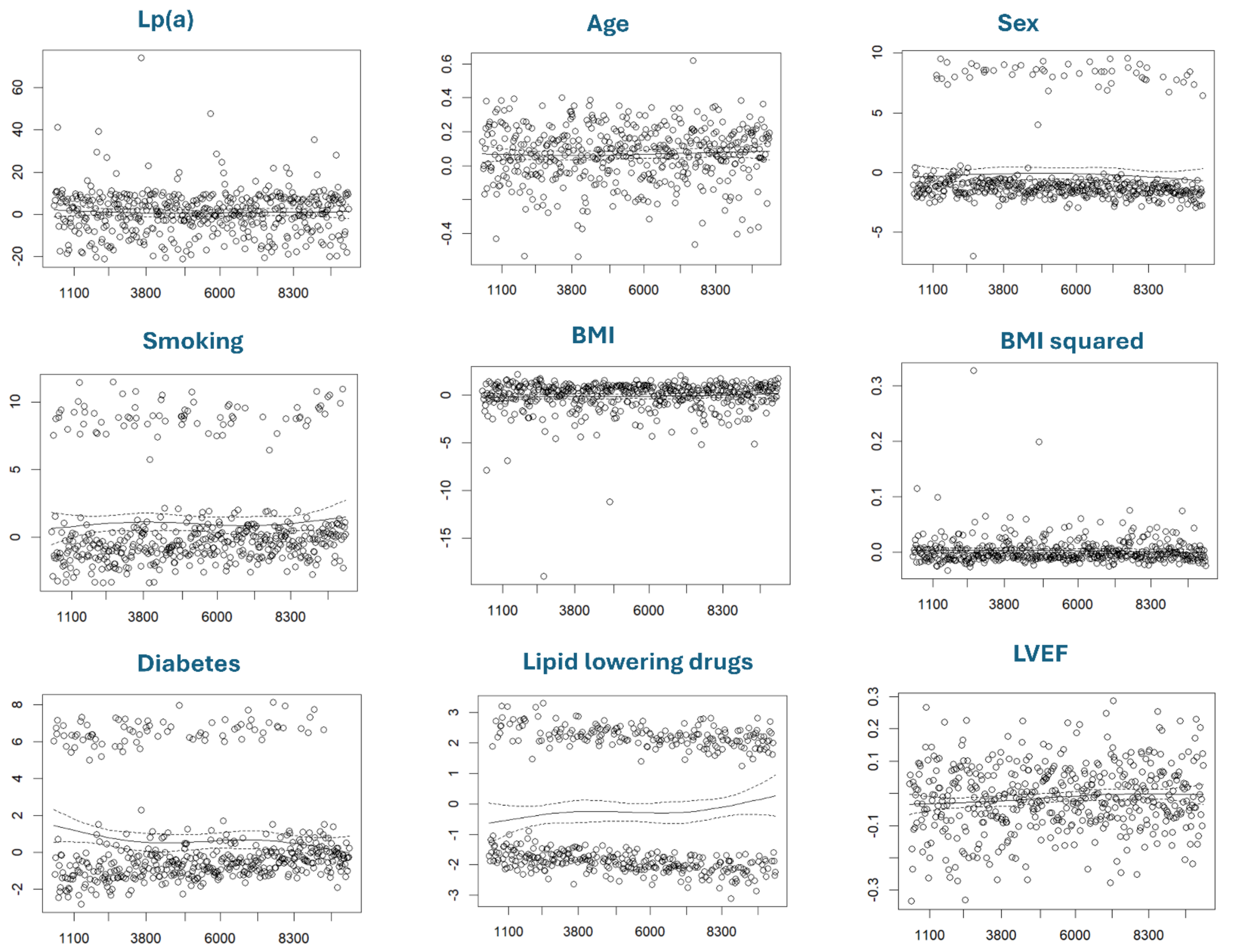

Global P value testing for correlation between Schoenfeld residuals and time was 0.080 and the p value for Lp(a) splines was 0.971.

Table SR2: Censoring date distribution for AMI patients. Red dots represent deaths, green dots represent censoring at the end of follow-up.

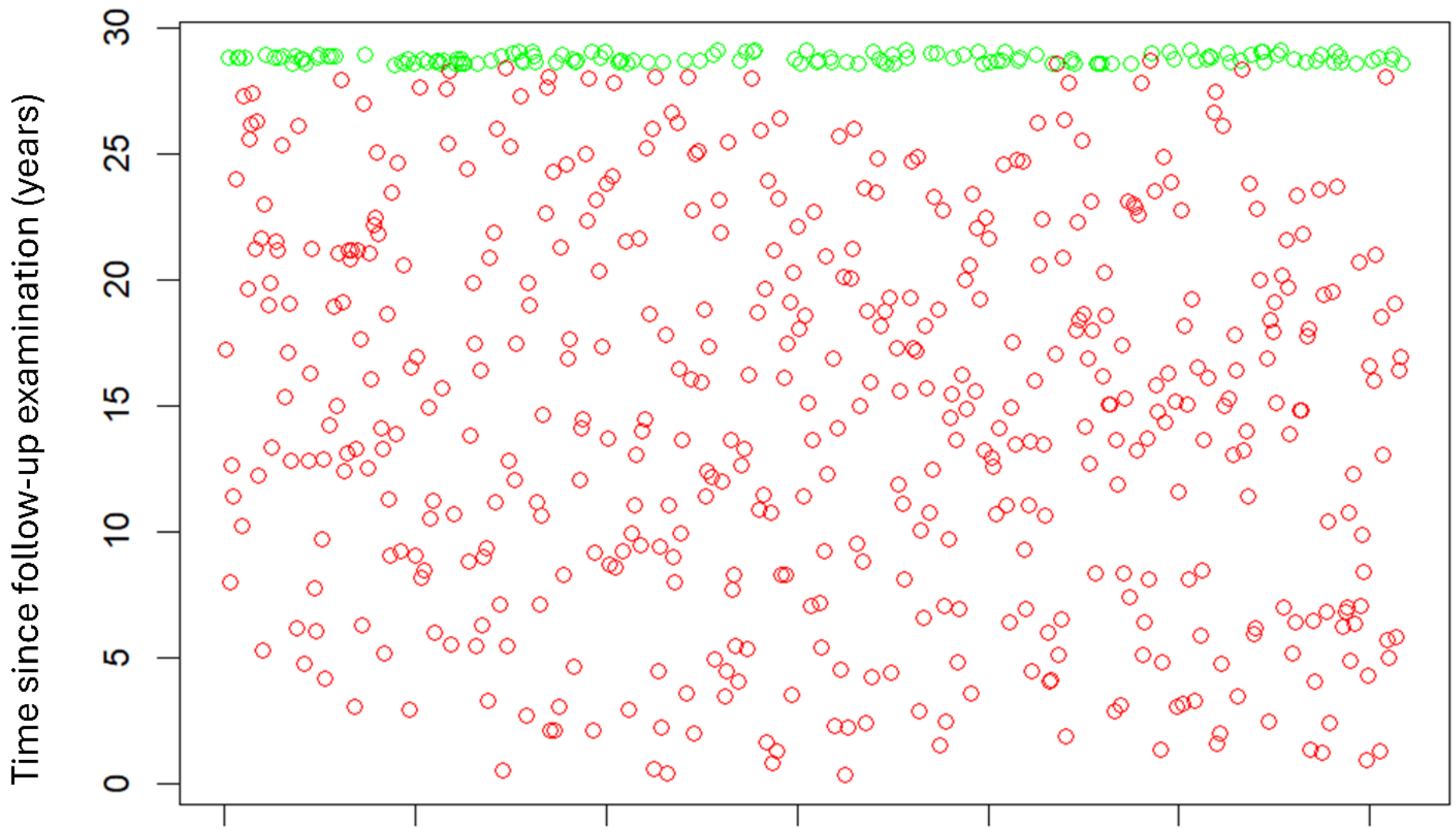

Table S1: Baseline characteristics of the total sample and stratified for myocardial infarction (no/yes). The group of individuals with prior myocardial infarction are further displayed stratified for Lp(a) high (≥ 50 mg/dL) and low (<50mg/dL).

	Total sample (N=1,186)			***AMI sample (N=641)***		
	Included (N=1104)	***Excluded* (N=82)***	***P-Value***	***Included (N=617)***	***Excluded* (N=24)***	***P Value***
Male	772 (69.9)	37 (45.1)	<0.001	538 (87.2)	18 (75.0)	0.155
Age (mean,SD)	55.9 (8.0)	53.5 (9.2)	0.023	56.6 (7.2)	53.0 (8.3)	0.045
Lp(a) (mg/dL)	15.0 (6.0 - 48.2)	16.5 (7.2 - 60.2)	0.240	17.0 (6.0 - 56.0)	17.0 (9.0 - 54.2)	0.413
Lp(a) ≥ 50mg/dL	273 (24.7)	26 (31.7)	0.203	167 (27.1)	8 (33.3)	0.658
BMI (kg/m²)	27.7 (25.4 - 30.3)	28.2 (25.3 - 31.2)	0.236	28.1 (25.8 - 30.6)	30.9 (28.4 - 31.3)	0.006
Diabetes mellitus	122 (11.1)	3 (3.7)	0.054	92 (15.0)	0 (0.0)	0.080
Smoking status			0.251			0.880
Never smoker	361 (32.8)	34 (41.5)		135 (22.0)	6 (25.0)	
Ex smoker	531 (48.2)	33 (40.2)		390 (63.4)	14 (58.3)	
Current smoker	210 (19.1)	15 (18.3)		90 (14.6)	4 (16.7)	
LVEF**	54.0 (46.0 - 61.0)	52.0 (46.0 - 60.8)	0.735	50.0 (44.0 - 58.0)	50.5 (45.8 - 58.5)	0.983
Medication at baseline						
Lipid lowering drugs	292 (26.5)	12 (14.6)	0.025	257 (41.7)	10 (41.7)	1
Statins	228 (20.7)	10 (12.2)	0.088	205 (33.2)	8 (33.3)	1
Beta blockers	453 (41.1)	26 (31.7)	0.121	394 (63.9)	17 (70.8)	0.630
Platelet aggregation inhibitors	550 (49.9)	23 (28.0)	<0.001	506 (82.0)	22 (91.7)	0.345
ACE inhibitors	219 (19.9)	6 (7.3)	0.008	171 (27.7)	2 (8.3)	0.062

* due to unknown survival status

** left-ventricular ejection fraction (Teichholz formula)

Table S2: Power calculations for different Hazard ratios and r^2 values

		**Hazard Ratio***		
		1.2	1.4	1.6
**r^2****	0.15	0.451	0.923	0.997
	0.25	0.407	0.890	0.993
	0.35	0.362	0.843	0.985
	0.45	0.315	0.779	0.968

* for a dichotomized Lp(a) variable
**r^2: The percent of variation in the predictor of interest explained by other covariates expected to be adjusted for in the Cox regression model

Assumptions: N=617 study participants, event probability: 0.775

R´s *pwr_coxph()* function (LucyMcGowan/survivalpwr) was used for the cacluations: *https://rdrr.io/github/LucyMcGowan/survivalpwr/man/pwr_coxph.html*

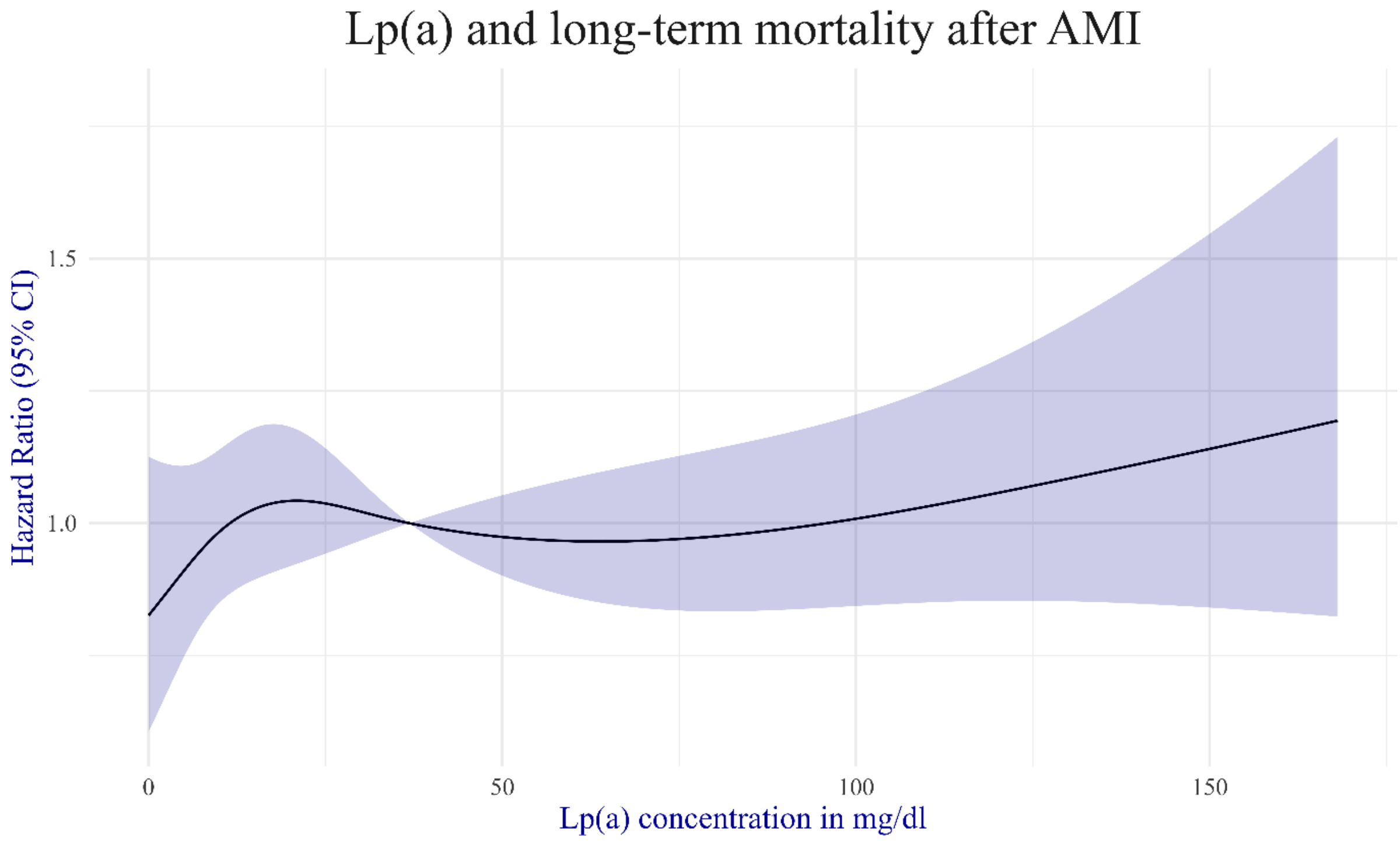

Figure S1: Results of the comprehensively adjusted Cox model analyzing the association between Lp(a) and long-term mortality. The model was adjusted for sex, age, BMI (linear and squared term), smoking, diabetes, hypertension, left-ventricular ejection fraction, LDL cholesterol, creatinine, and intake of lipid lowering drugs.

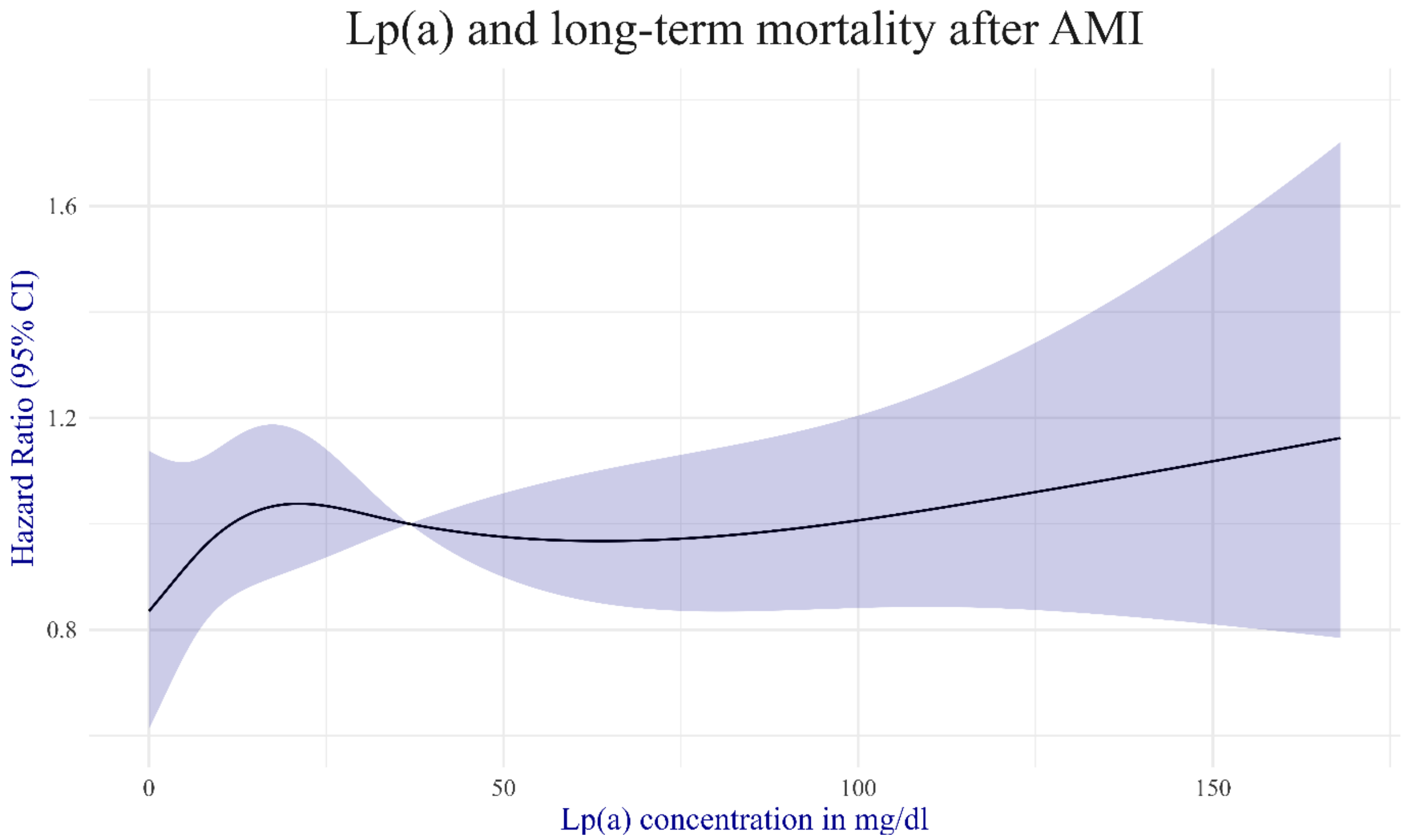

Figure S2: Association between Lp(a) and long-term mortality in the AMI group. Patients with death within two years after follow-up examination (N = 17) were excluded.

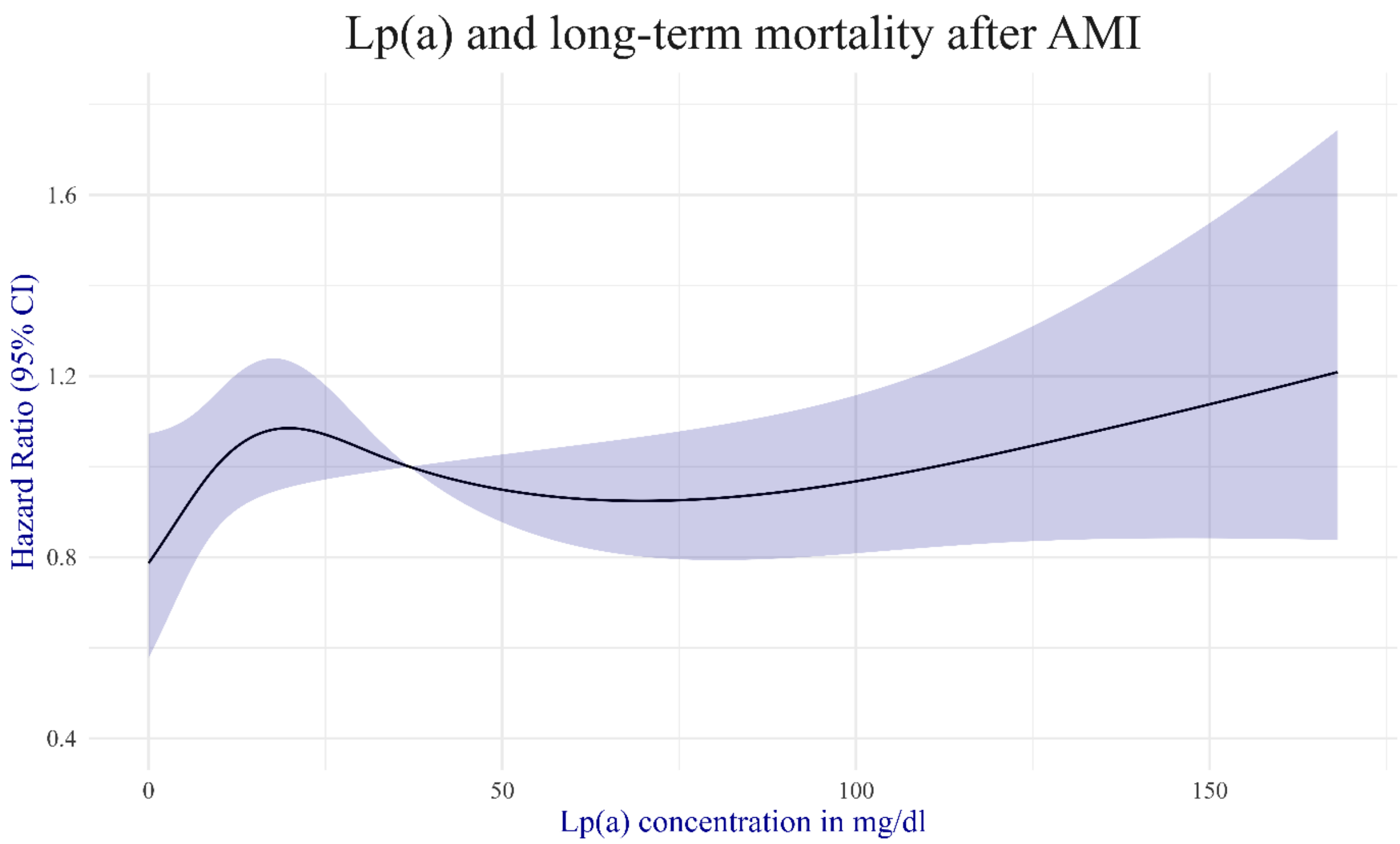

Figure S3: Association between Lp(a) and long-term mortality in the AMI group using age as timescale to address left-truncating of data.

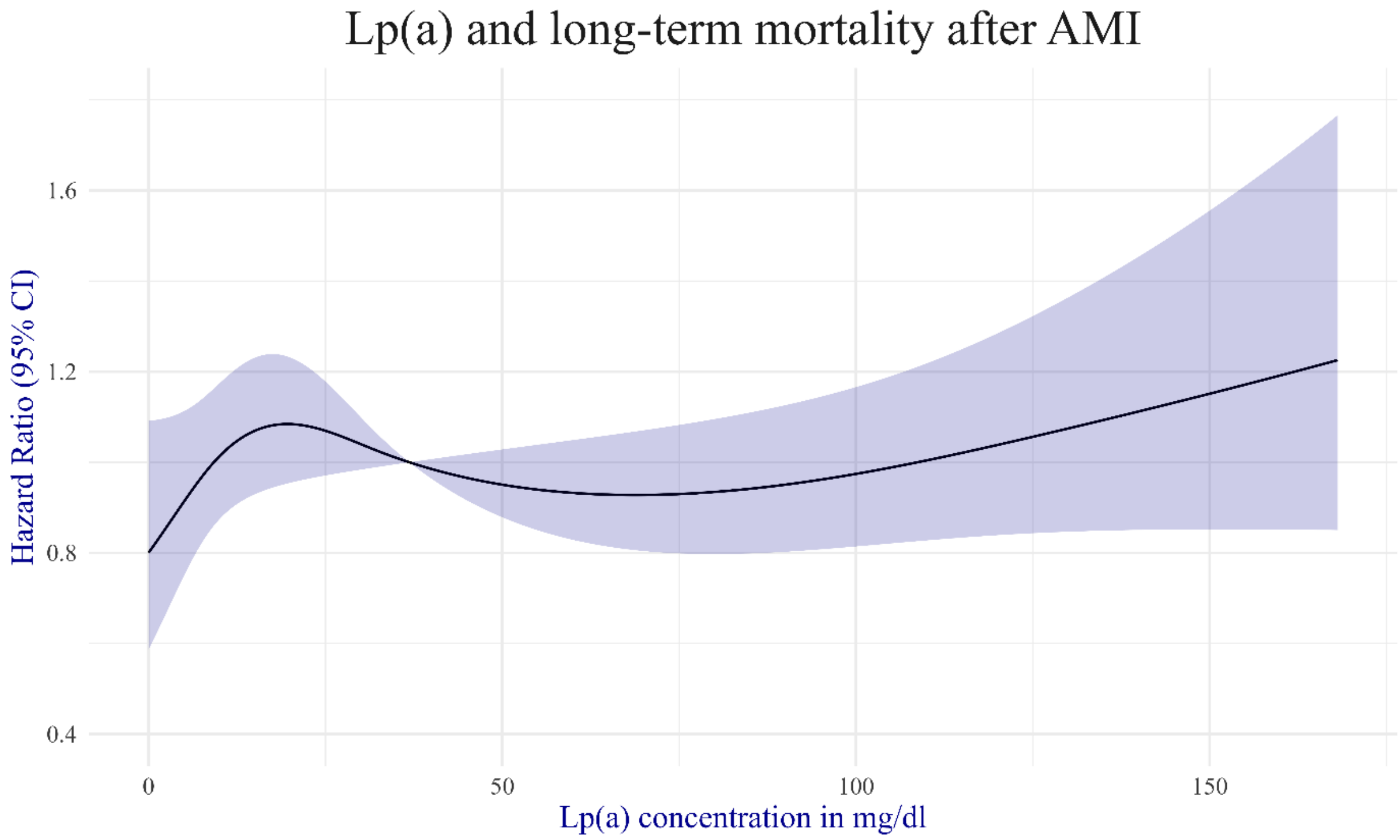

Figure S4: Association between Lp(a) and long-term mortality in the AMI group by calculating time-since-AMI delayed-entry Cox model to address left-truncating of data.

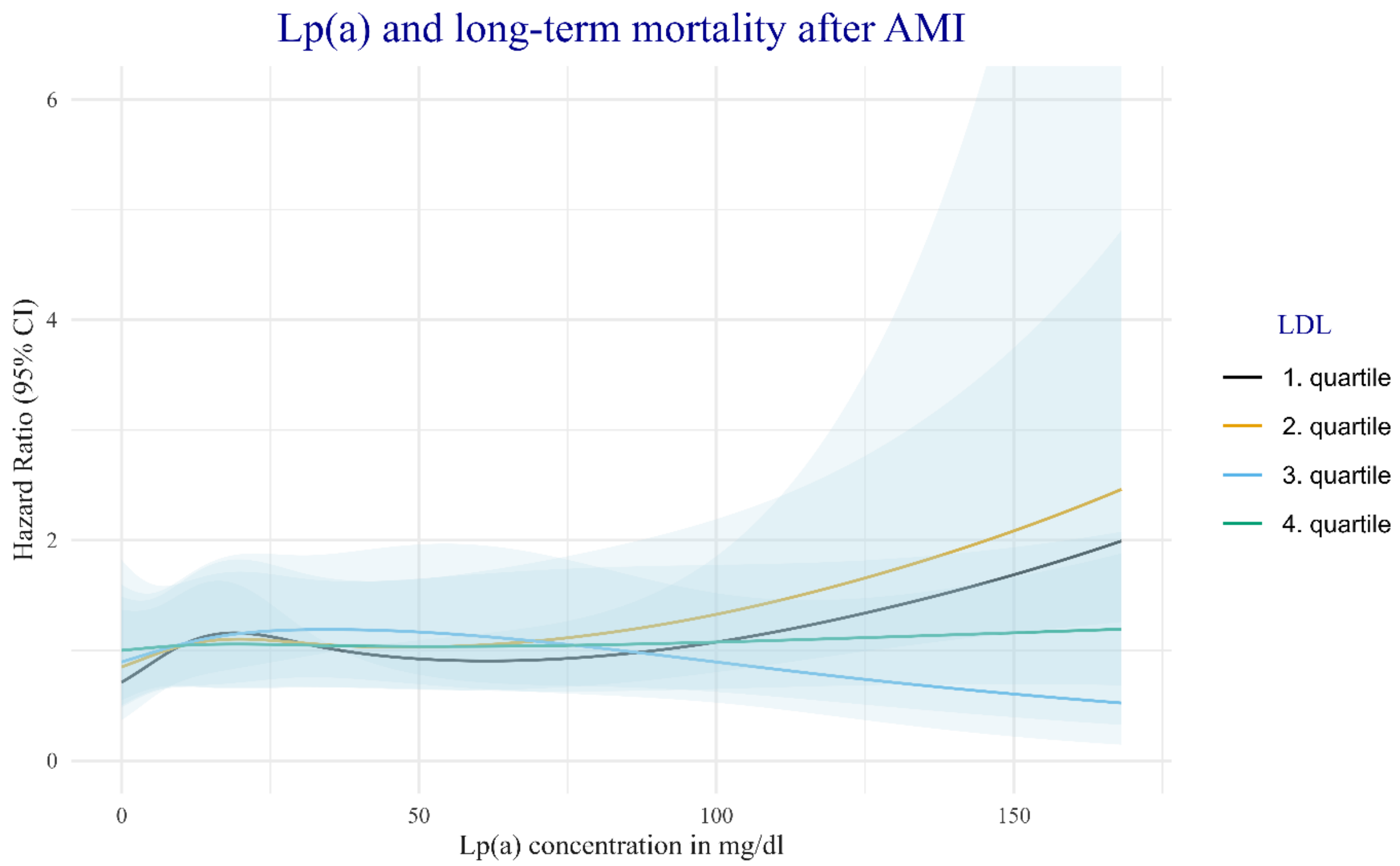

Figure S5: Interaction between Lp(a) concentrations (X axis) and quartiles of LDL (Low-density lipoprotein) concentrations.

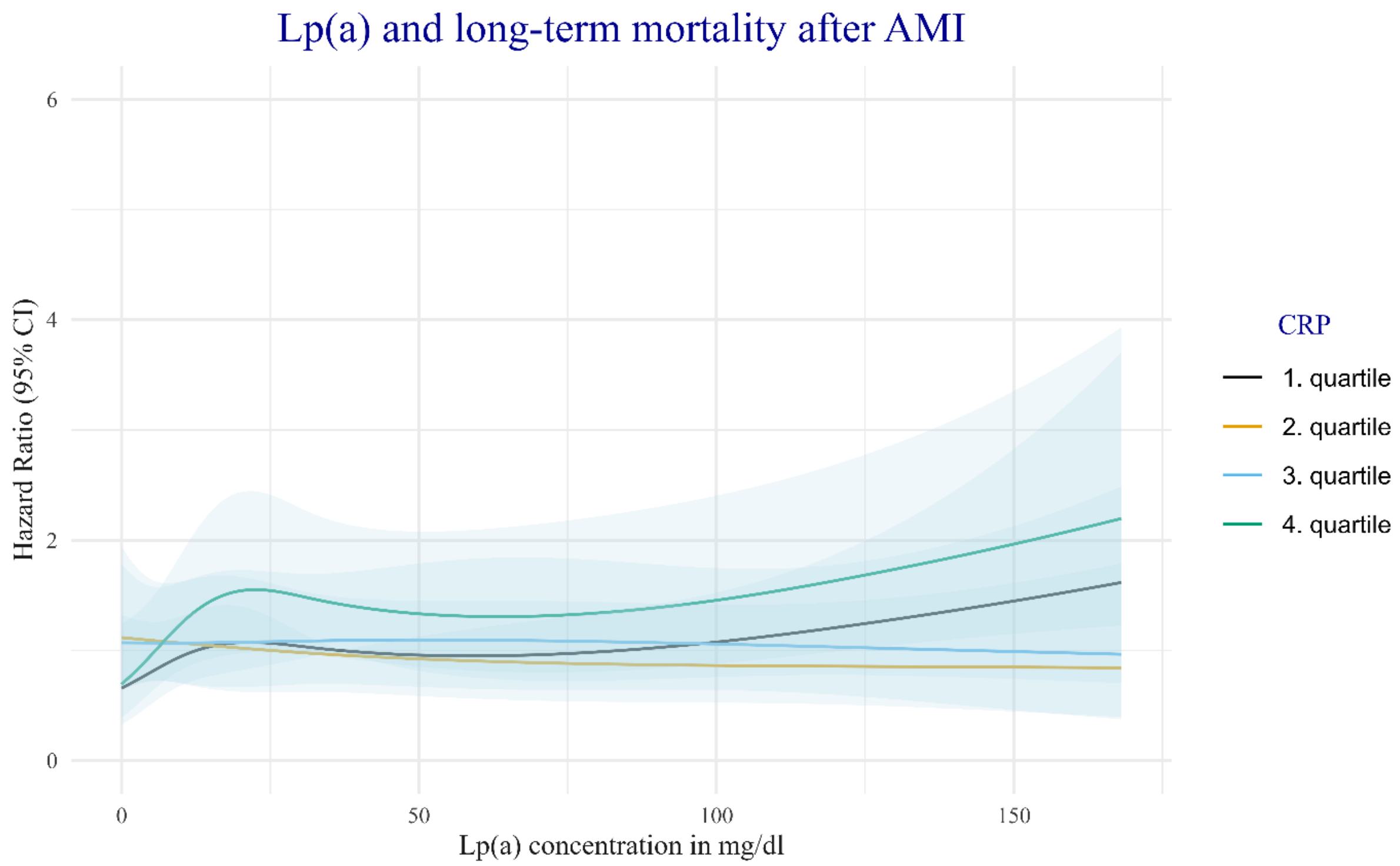

Figure S6: Interaction between Lp(a) concentrations (X axis) and quartiles of CRP (c-reactive protein) concentrations.

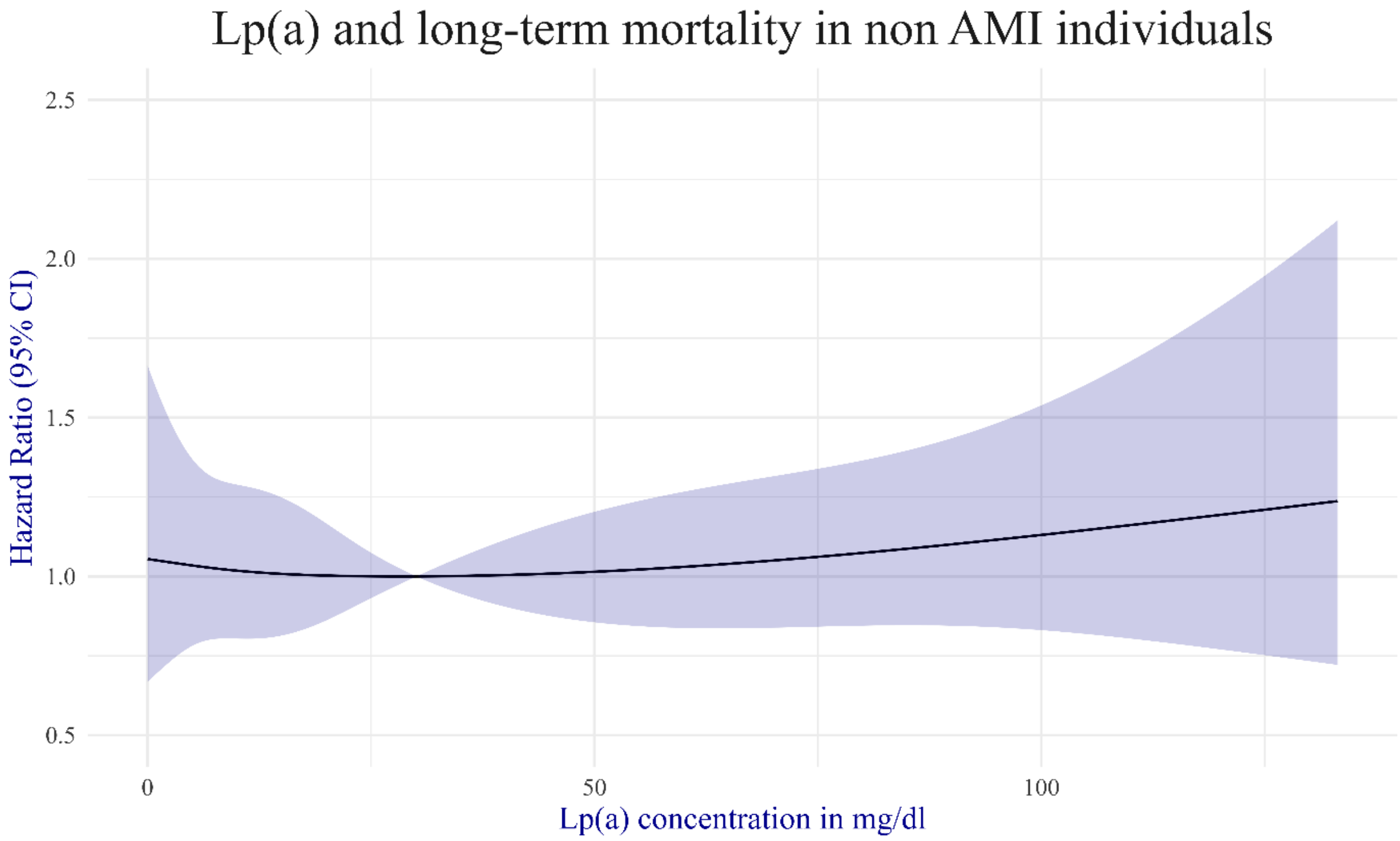

Figure S7: Results of the Cox model including only the siblings without a history of hospitalized AMI. The association between Lp(a) and long-term mortality was modeled using restricted cubic splines with 4 knots.

Supplementary text 1: Interaction analysis

Results of the Cox model including multiplicative interactions terms for Lp(a) [restricted cubic splines] and diabetes (categorical), LDL (continuous) and CRP (continuous) respectively.

	**ß coefficient**	**p values**
Diabetes*Lp(a)´	0.0249	0.507
Diabetes*Lp(a)´´	-0.6093	0.556
Diabetes *Lp(a)´´´	0.7927	0.576
LDL*Lp(a)´	-0.0005	0.249
LDL*Lp(a)´´	0.0109	0.305
LDL*Lp(a)´´´	-0.0150	0.305
CRP*Lp(a)´	0.0064	0.012
CRP *Lp(a)´´	-0.1321	0.050
CRP *Lp(a)´´´	0.1765	0.057

Results of the aalen additive hazards models for the interaction terms between natural splines for Lp(a) and continuous LDL and CRP respectively.

	**coefficient**	**p values**
LDL*Lp(a)´	-3.53e-07	0.616
LDL*Lp(a)´´	-3.49e-07	0.658
LDL*Lp(a)´´´	-9.56e-07	0.373
LDL*Lp(a)´´´´	-7.35e-08	0.957
LDL*Lp(a)´´´´´	4.81e-07	0.534
CRP*Lp(a)´	8.82e-06	0.342
CRP *Lp(a)´´	5.44e-06	0.538
CRP *Lp(a)´´´	-3.79e-06	0.710
CRP *Lp(a)´´´´	-7.35e-06	0.738
CRP *Lp(a)´´´´´	1.32e-05	0.711

RERI values

- Lp(a)*LDL: -0.12 (-0.58; 0.33), p value: 0.705
- Lp(a)*CRP: 0.12 (-0.34;0.58), p value: 0.308
